# Estimation of the Acoustic Properties of the Random Packing Structures of Granular Materials: Estimation of the Sound Absorption Coefficient Based on Micro-CT Scan Data

**DOI:** 10.3390/ma16010337

**Published:** 2022-12-29

**Authors:** Shuichi Sakamoto, Kyosuke Suzuki, Kentaro Toda, Shotaro Seino

**Affiliations:** 1 Department of Engineering, Niigata University, Ikarashi 2-no-cho 8050, Nishi-ku, Niigata 950-2181, Japan; 2 Graduate School of Science and Technology, Niigata University, Ikarashi 2-no-cho 8050, Nishi-ku, Niigata 950-2181, Japan

**Keywords:** granular material, random packing, micro-CT scan, tortuosity

## Abstract

In this study, the sound absorption properties of randomly packed granular materials were estimated. Generally, it is difficult to construct a general mathematical model for the arrangement of randomly packed granular materials. Therefore, in this study, an attempt was made to estimate the sound absorption coefficient using a theoretical analysis by introducing data from computed tomography (CT) scans, as the tomographic images of CT scans correspond to the slicing and elemental division of packing structures. In the theoretical analysis, the propagation constants and characteristic impedances in the voids were obtained by approximating each tomographic image as a void between two parallel planes. The derived propagation constants and characteristic impedances were then treated as a one-dimensional transfer matrix in the propagation of sound waves, and the transfer matrix method was used to calculate the normal incident sound absorption coefficient. The theoretical value of the sound absorption coefficient was derived using the effective density to which the measured tortuosity was applied. As a result, for the theoretical values considering the tortuosity, in many cases, the theoretical values were close to the measured values. For the theoretical values, when both the surface area and tortuosity were considered, the peak sound absorption frequency moved to a lower frequency and was in general agreement with the measured values.

## 1. Introduction

Granular packing structures have been used for noise reduction purposes, such as low-noise pavements [1], ballasted tracks [2] and consolidated expanded clay granulates [3], due to their acoustic properties. These structures have continuous voids and exhibit acoustic properties based on the same principle of porous materials, and their acoustic properties vary depending on their layer thickness, grain size, and filling structure. Therefore, from an engineering point of view, it is useful to predict the acoustic properties of granular packing structures through calculations based on their grain size, packing structure, and gas physical properties.

Various studies have been performed on the sound absorption properties of the regular packing structures of granular materials. For example, while there are experimental studies on the acoustic properties of loosely packed lattices of granular materials [4], predicting the acoustic properties of face-centered cubic lattices [5], and fundamental studies on the acoustic properties of granular materials [6], such as sound absorption by simple cubic and hexagonal lattices [7] and by hexagonal most dense and face-centered cubic lattices [8], there are few examples of theoretical calculations on random packing, although experimental studies [9] have been previously performed. Additionally, numerical analyses have been performed on several regular packing structures using commercial software [10] (e.g., a numerical analysis of pseudo-random packing combined with multiple regular packings) [11] and on the sound absorption characteristics of random close packing using the discrete element method (DEM) [12]. However, the software used for the theoretical analyses in these studies is expensive and computationally time-consuming. Generally, when granular materials are packed, the structure is random packing. Therefore, it is scientifically significant to analyze random packing rather than regular packing.

In this study, the sound absorption properties of randomly packed granular materials were estimated. Generally, it is difficult to construct a general mathematical model for the arrangement of randomly packed granular materials. Therefore, in this study, an attempt was made to estimate the sound absorption coefficient using a theoretical analysis by introducing data from computed tomography (CT) scans, as the tomographic images of CT scans correspond to the slicing and elemental division of packing structures. To estimate the propagation constants and characteristic impedances of voids, we considered each tomographic image as a void between two parallel planes using theoretical calculations. The transfer matrix method was used to calculate the normal incident sound absorption coefficient, which was estimated from the propagation constants and characteristic impedances generated from sound waves travelling through the medium. This method allows the sound absorption coefficient to be calculated based on CT scan images of granular materials. Moreover, the used program is simple, the calculation time is short, and the calculation can be performed using a common PC. By applying this method, it may be possible to calculate the sound absorption coefficients of granular sound-absorbing materials with irregular shapes, not limited to the glass beads used in this measurement.

Tortuosity, one of the Biot parameters, is a parameter that expresses the complexity of the sound waves propagating in a structure, and it was measured in this study. As a result, by combining the measured tortuosity with an effective density, it is possible to calculate the theoretical value of the sound absorption coefficient.

Regarding the measurement of the sound absorption coefficient, a two-microphone impedance tube was used to measure the normal incident sound absorption coefficient of each sample. The results of a comparison between the experimental measurements and theoretical values were also reported.

## 2. Samples and Measuring Equipment Used for Measuring the Sound Absorption Coefficient

### 2.1. Sample for Measuring the Sound Absorption Coefficient

**Figure 1:**
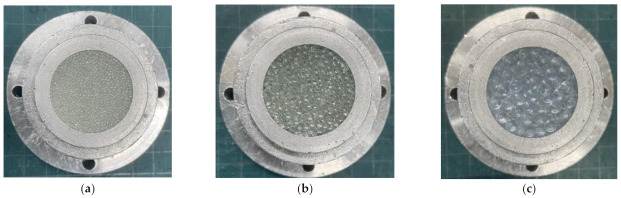
Test samples of random packing granules: (**a**) *d* = 1 mm; (**b**) *d* = 2 mm; (**c**) *d* = 4 mm.

In this study, random packing was used as a packing structure for granular materials. Figure 1a–c shows the samples used to measure the sound absorption coefficient. Glass beads with diameters of 1 mm, 2 mm, and 4 mm were used in the experiments.

A sample holder tube by aluminum alloy with an inner diameter of 29 mm was filled with glass beads.

### 2.2. Equipment for Measuring the Sound Absorption Coefficient

**Figure 2:**
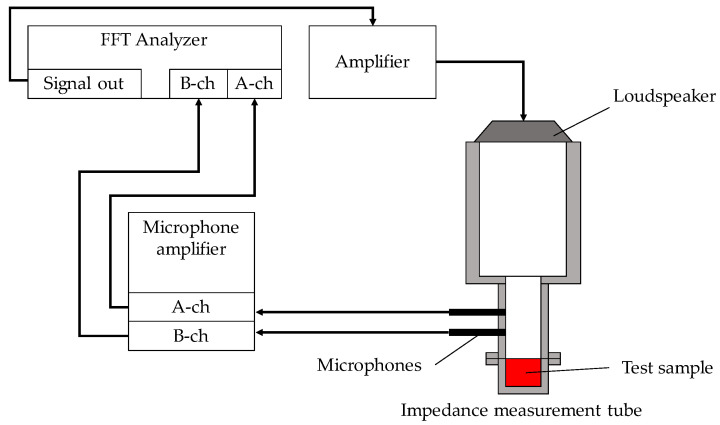
Scheme of two microphone impedance tubes for the sound absorption coefficient measurement.

A Brüel & Kjær Type 4206 two-microphone impedance tube (Brüel & Kjær Sound & Vibration Measurement A/S, Skodsborgvej 307 DK-2850, Nærum, Denmark) was used to measure the sound absorption coefficient, and Figure 2 shows the configuration of the apparatus. As shown in the figure, a sample was enclosed in the impedance tube, a sinusoidal signal was output by the signal generator DS-3000 with a built-in fast Fourier transform (FFT) analyzer fabricated by Ono Sokki (Yokohama, Japan), and the transfer function between the sound pressure signals of the two microphones attached to the impedance tube was measured by the FFT analyzer. The measured transfer function was used to calculate the normal incident sound absorption coefficient in accordance with ISO 10534-2. The critical frequency for the formation of plane waves differs based on the inner diameter of the acoustic tube. Small tubes with an inner diameter of 29 mm were used because the sound absorption coefficient in the low-frequency range was not high enough for the samples used in this study. Therefore, the measurement range was 500–6400 Hz.

## 3. Methods and Results of Measuring Tortuosity

### 3.1. Tortuosity Measurement Sample

**Figure 3:**
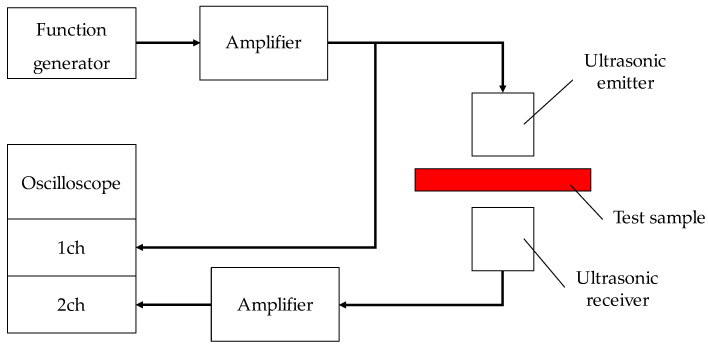
Configuration diagram of measurement of tortuosity.

As indicated in the previous report and in Figure 3, tortuosity is one of the Biot parameters [8]. In this study, the tortuosity of randomly packed structures was considered and was experimentally measured using ultrasonic sensors. In general, the tortuosity *α*_∞_ can be expressed using the speed of sound *c*_0_ in air and the apparent speed of sound *c* in the filling structure, as shown in Equation (1) [13]:

(1) $$\alpha_{\infty }={\left(\frac{c_{0}}{c}\right)}^{2}$$

### 3.2. Tortuosity Measurement

Tortuosity was measured using the same measuring equipment as previously reported [8]. Glass beads with a grain size of *d* = 2 mm were used for the measurements, as the tortuosity is independent of grain size.

Ultrasonic sensors with center frequencies of 32.7 kHz, 58 kHz, 110 kHz, 150 kHz, 200 kHz, and 300 kHz were used. In the initial phase, we measured the tortuosity α∞ at each frequency to determine the performance of the random packing structure. The inverse of the square root of the frequency used for the measurement was then taken as the value on the horizontal axis, and the tortuosity α∞ at each frequency (determined by the meas-urement) was plotted as the value on the vertical axis. A linear approximation of these points was performed using the least-squares method, yielding a straight line that rose steadily to the right. The extreme value of the tortuosity at infinite frequency in the approximated straight line was obtained, where the *y*-intercept of the graph was the tortuosity *α*_∞_ of the packing structure.

The signal-to-noise ratio (S/N ratio) of the ultrasonic sensor was low at high frequencies due to the low conversion efficiency and high sound wave attenuation. A total of 150 measurements were synchronously added to increase the S/N ratio and improve the measurement accuracy. The signals were measured with a resolution of 16 bits.

**Figure 4:**
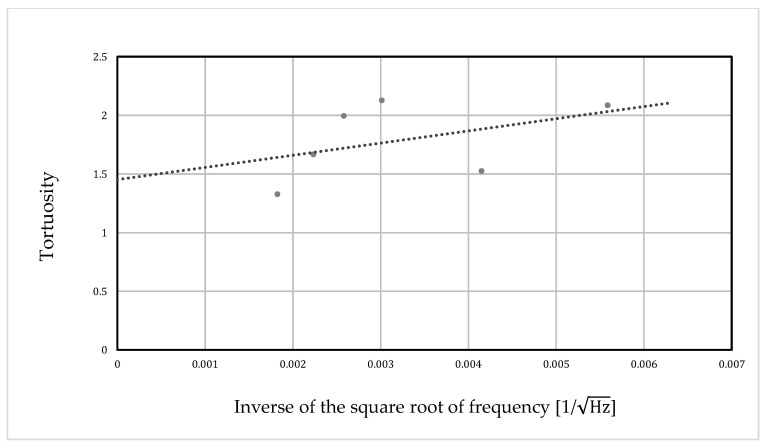
Results of the tortuosity measurements.

Table 1 shows the results of the tortuosity measurements, and Figure 4 shows a graphical representation. This was confirmed by the fact that the y-intercept of the approximation line indicated the complexity of each packing configuration. The measurement results show that the tortuosity was *α*_∞_ = 1.45.

## 4. Theoretical Analysis

### 4.1. Analysis Unit and Element Division of the Packing Structure

In random packing, there is no periodicity in the arrangement of granular materials, making it difficult to develop mathematical models. Therefore, this study considered the use of geometric information on the measured particles, and the method adopted for this purpose was micro-CT scanning.

**Figure 5:**
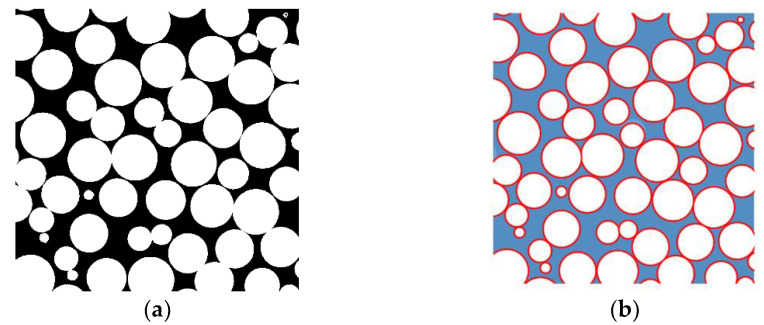

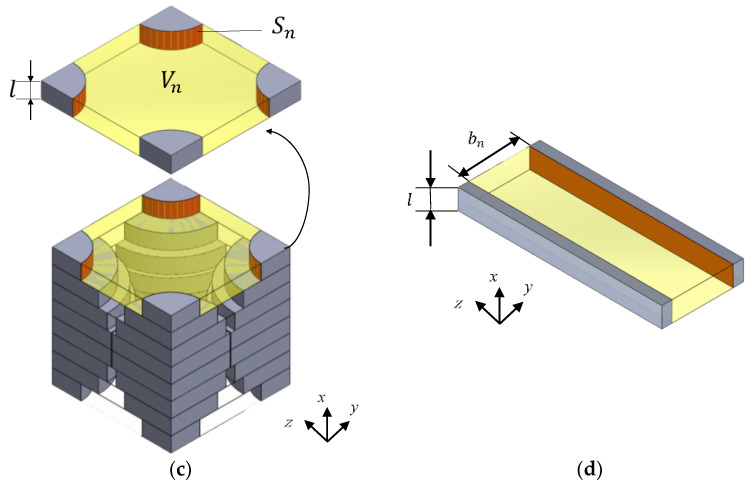
Divided element approximated to the clearance between the two planes: (**a**) binarization of the cross-sectional image; (**b**) calculation of the circumference of the sphere and the cross-sectional area of the clearance; (**c**) divided element; (**d**) approximated clearance between the two planes.

The cross-sectional image was binarized, as shown in Figure 5a. The sphere boundaries were then clarified, as shown in Figure 5b. The cross-sectional images of the micro-CT scan were taken over a rectangular area of 20 mm in the *x*-direction and 13 mm square in the *y*-*z* plane. The instrument used was an MCT225 Metrology CT, manufactured by NIKON Corp (Tokyo, Japan). The tomograms were taken to be sliced in the *y*-*z* plane, which was perpendicular to the direction of the sound wave incidence (*x*-direction). The CT scan data for *d* = 2 mm were also used for the analysis of other grain sizes, as the arrangement of grains was independent of the grain size.

As shown in Figure 5c, the cross-section of the sphere in the tomogram was approximated to a cylindrical shape of length *l*. Subsequently, as shown in Figure 5d, it was approximated as a clearance between two planes, where the surface area *S_n_* of the grain and the volume *V*_n_ of the clearance were equal. The number of images used for the theoretical analysis *n* was 2000, 1000, and 500 for the grain size *d* = 1 mm, *d* = 2 mm, and *d* = 4 mm, respectively, for 20 mm in the *x*-direction. Thus, the thickness *l* of the element in Figure 5c corresponded to the pitch in the *x*-direction of the image, which was 10 µm, 20 µm, and 40 µm for the grain sizes of *d* = 1 mm, 2 mm, and 4 mm, respectively. Thus, for each grain size *d* = 1 mm, 2 mm, and 4 mm, the number of analysis units was *n* = 100 in the packing structure partitioning method [7,8], as shown in Figure 5c, which was a value at which the theoretical values of the normal incident sound absorption coefficient sufficiently converged.

For each image, the cross-sectional area of the void and the sum of the circumferences of the cross-sections of all of the spheres in the image were calculated. Multiplying the cross-sectional area of the void by the image pitch *l* provided the volume of the void *V*_n_, as shown in Figure 5c. Similarly, multiplying the total circumference of the cross-section by *l* yielded *S_n_*, as shown in Figure 5c. Thus, as shown in Figure 5d, using Equation (2), the thickness of the clearance between the two planes, *b*_n_, could be obtained for a single image with a thickness *l*. Here, *F*_n_ is the correction factor for obtaining the true surface area, explained in the next section.

(2) $$b_{n}=\frac{2V_{n}}{F_{n}S_{n}}$$

### 4.2. Numerical Analysis of the Tomograms in Random Packing

**Figure 6:**
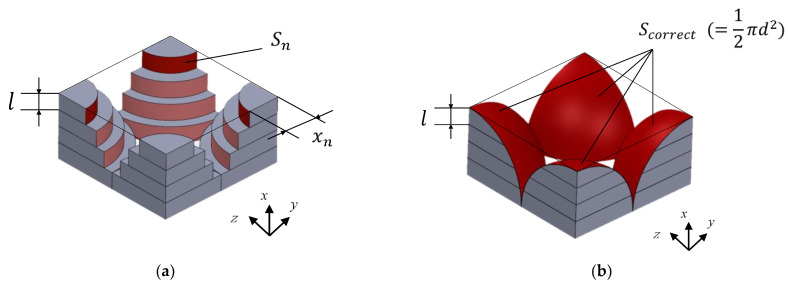
Divided element for the half sphere: (**a**) cylindrically approximated surface area; (**b**) true surface area.

Using the method shown in Figure 6a, the surface area of the granular material used in the analysis was estimated to be smaller than the surface area of a true sphere. Thus, a correction for the surface area was made, as shown in Figure 6b and below.

In Figure 6a, the radius *x*_n_ of the cylindrical element in the *n*th division is expressed as in Equation (3), where *k* is the number of divisions of the hemisphere in the *x*-direction.

(3) $$x_{n}=\sqrt{\frac{d^{2}}{4}-{\left\{\left(n-1\right)\frac{d}{2k}\right\}}^{2}}$$

Using the above equation, the area *S_n_* of the cylindrical element could be expressed as in Equation (4):

(4) $$S_{n}=\pi x_{n}\frac{d}{k}$$

When the number of divisions *k* = 1, the ratio between the sum of the areas *S_n_* of the divided cylindrical elements obtained from the CT images and the true surface area *S_correct_* (= 1/2·*πd*^2^) of the hemisphere is shown in the following equation, where *F*_1_ is the correction factor.

(5) $$F_{1}=\frac{S_{correct}}{\underset{k\to 1}{\lim}\sum_{n=1}^{k}S_{n}}=1$$

As shown in the above equation, it is geometrically clear that when the number of divisions *k* = 1, the correction factor *F*_1_ is unity (i.e., they are equal). However, when the number of divisions *k* is infinite, the correction factor *F*_∞_ asymptotically approaches ~1.273.

(6) $$F_{\infty }=\frac{S_{correct}}{\underset{k\to \infty }{\lim}\sum_{n=1}^{k}S_{n}}≅1.273$$

Furthermore, the correction factor *F*_50_ for *k* = 50 is ~1.259.

(7) $$F_{50}=\frac{S_{correct}}{\underset{k\to 50}{\lim}\sum_{n=1}^{k}S_{n}}≅1.259$$

Therefore, for the resolution of the CT scan used in this study, an area equal to the surface area of the true sphere could be used for the analysis by multiplying the correction factor *F*_50_ = 1.259 for *k* = 50 by *S_n_* in Equation (2).

### 4.3. Propagation Constants and Characteristic Impedance Considering the Tortuosity

**Figure 7:**
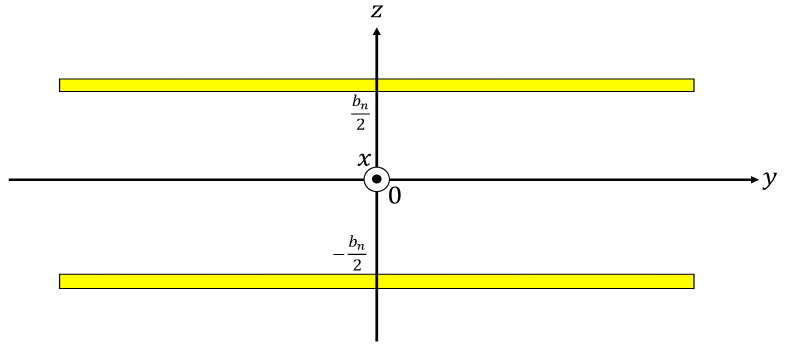
Coordinates for the clearance between the two planes.

Our investigation was based on Stinson [14] and Allard [15] descriptions of investigative procedures. The Cartesian coordinate system was used, as shown in Figure 7, and through a three-dimensional analysis using the Navier–Stokes equations, the gas equation of state, continuity equation, energy equation, and dissipative function representing the heat transfer, effective density *ρ_s_*, and compressibility *C_s_* were derived, as shown in Equations (8) and (9), respectively [14]. For several atmospheric properties, including the density (*ρ*_0_) of air, *λ_s_* is the parameter of mediation, *b_n_* is the clearance thickness between the two planes, *ω* is the angular frequency, *η* is the viscosity of air, *κ* is the specific heat ratio of air, *P*_0_ is the atmospheric pressure, and *N_pr_* is the Prandtl number.

(8) $$\rho_{s}=\rho_{0}{\left[1-\frac{\tanh\left(\sqrt{j}\lambda_{s}\right)}{\sqrt{j}\lambda_{s}}\right]}^{-1},\lambda_{s}=\frac{b_{n}}{2}\sqrt{\frac{\omega \rho_{0}}{\eta }}$$

(9) $$C_{s}=\left(\frac{1}{\kappa P_{0}}\right)\left\{1+\left(\kappa -1\right)\left[\frac{\tanh\left(\sqrt{jN_{pr}}\lambda_{s}\right)}{\sqrt{jN_{pr}}\lambda_{s}}\right]\right\}$$

Using the effective density *ρ_s_* multiplied by the tortuosity *α_∞_*, the propagation constant and characteristic impedance considering the tortuosity could be obtained [15]. Therefore, the propagation constant *γ* and characteristic impedance *Z_c_* when considering the tortuosity *α_∞_* could be expressed using the effective density *ρ_s_* and compression ratio *C_s_*, as follows [15]:

(10) $$\gamma =j\omega \sqrt{\alpha_{\infty }\rho_{s}C_{s}}$$

(11) $$Z_{c}=\sqrt{\frac{\alpha_{\infty }\rho_{s}}{C_{s}}}$$

### 4.4. Transfer Matrix

**Figure 8:**
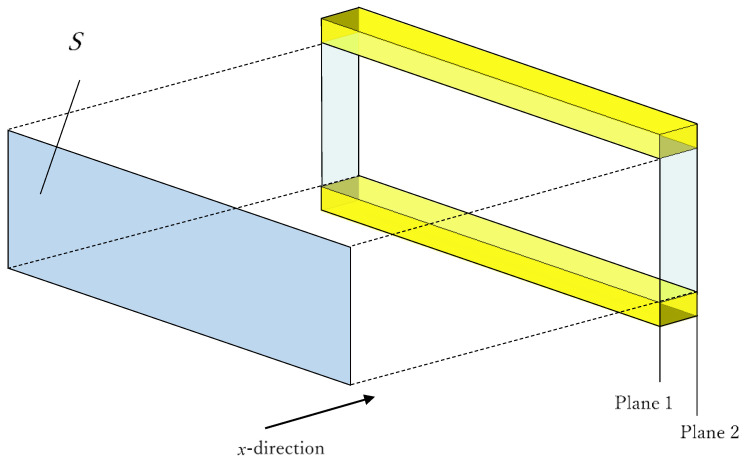
Cross-sectional shape at any position of the analysis unit (sound incident area and aperture area).

Using a one-dimensional wave equation, the transfer matrix method was used to derive the clearance between the two planes for the sound pressure and volume velocity. Figure 8 shows a schematic representation of one component of the *x*-direction clearance between the two planes indicated in Figure 7. Using the characteristic impedance, propagation constants, and cross-sectional area *S* of the clearance, as well as the length *l* of the divided element derived in the previous section, the transfer matrix *T_n_* and four-terminal constants *A*~*D* of the acoustic tube element could be calculated, as shown in Equation (12):

(12) $$T_{n}=\left[\begin{array}{cc} \cosh\left(\gamma l\right) & \frac{Z_{c}}{S}sinh\left(\gamma l\right)\\ \frac{S}{Z_{c}}sinh\left(\gamma l\right) & \cosh\left(\gamma l\right) \end{array}\right]=\left[\begin{array}{cc} A & B\\ C & D \end{array}\right]$$

Plane 1 is the plane of incidence of the sound wave, and Plane 2 is the plane of transmission of the sound wave. If the sound pressure and particle velocity at Plane 1 are *p*_1_ and *u*_1_, respectively, and the sound pressure and particle velocity at Plane 2 are *p*_2_ and *u*_2_, respectively, the transfer matrix can be expressed as in Equation (13):

(13) $$\left[\begin{array}{c} p_{1}\\ Su_{1} \end{array}\right]=\left[\begin{array}{cc} A & B\\ C & D \end{array}\right]\left[\begin{array}{c} p_{2}\\ Su_{2} \end{array}\right]$$

**Figure 9:**
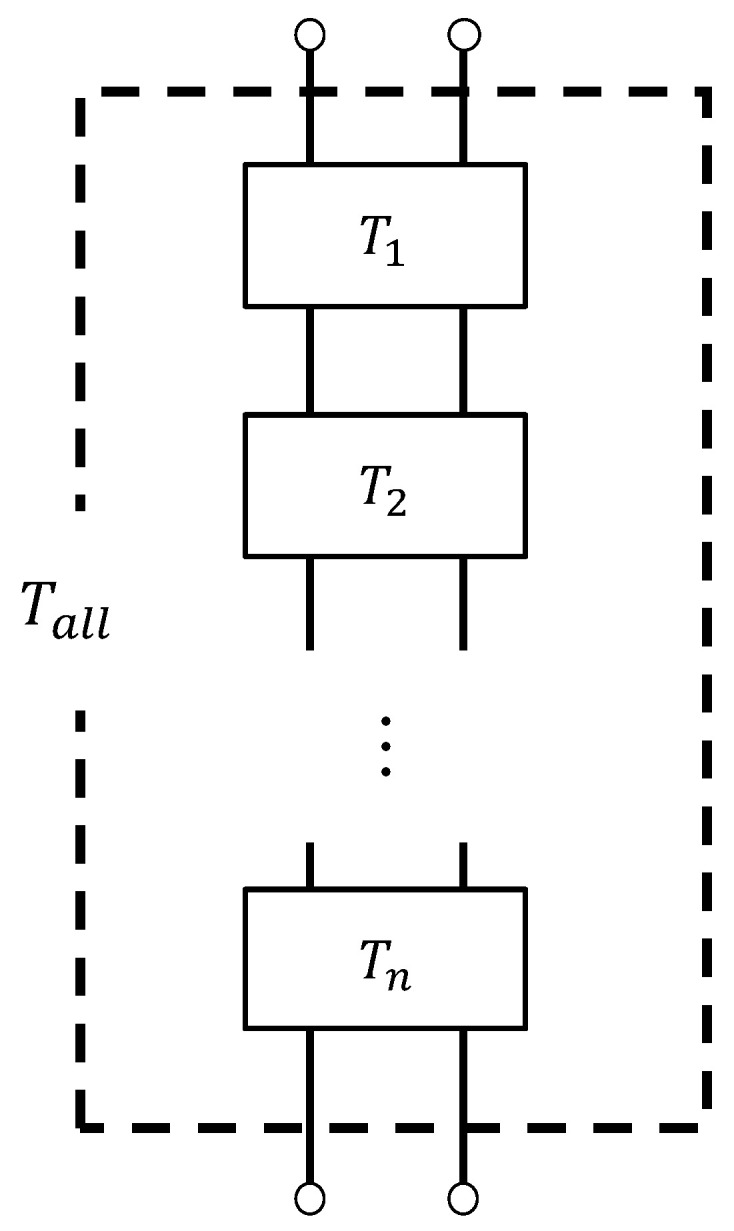
Equivalent circuit in the analysis (cascade connection of the transfer matrix of each element).

As shown in Figure 9, an equivalent circuit was used to construct the transfer matrices *T*_unit_ and *T*_top_ for the analysis unit at the top end of the sample, respectively, by cascading the transfer matrices of each divided element.

### 4.5. Vertical Incident Sound Absorption Coefficient

The transfer matrix, described in Section 4.4, was used to calculate the sound absorption coefficient. Since the end of the sample used in this study was a rigid wall, the particle velocity *u*_2_ = 0, and Equation (13) could be transformed into Equation (14):

(14) $$\left[\begin{array}{c} p_{1}\\ Su_{1} \end{array}\right]=\left[\begin{array}{cc} A & B\\ C & D \end{array}\right]\left[\begin{array}{c} p_{2}\\ 0 \end{array}\right]=\left[\begin{array}{c} Ap_{2}\\ Cp_{2} \end{array}\right]$$

If the sound pressure and particle velocity immediately outside Plane 1 are denoted by *p*_0_ and *u*_0_, respectively, the specific acoustic impedance *Z*_0_ looking inward from the plane of incidence of the sample can be expressed as follows:

(15) $$Z_{0}=\frac{p_{0}}{u_{0}}$$

Therefore, through *p*_0_ = *p*_1_ and Equation (15), the specific acoustic impedance *Z*_0_ of the sample can be expressed as follows:

(16) $$Z_{0}=\frac{p_{0}}{u_{0}}=\frac{A}{C}S$$

The relationship between the specific acoustic impedance *Z*_0_ and reflectance *R* can be expressed as follows:

(17) $$R=\frac{Z_{0}-\rho_{0}c_{0}}{Z_{0}+\rho_{0}c_{0}}$$

The following relationship between the sound absorption coefficient, the reflectance, and Equation (17) provides the theoretical value of the sample’s normal incident sound absorption coefficient *α*.

(18) $$\alpha =1-{\left|R\right|}^{2}$$

## 5. Comparison of the Measured and Theoretical Values

**Figure 10:**
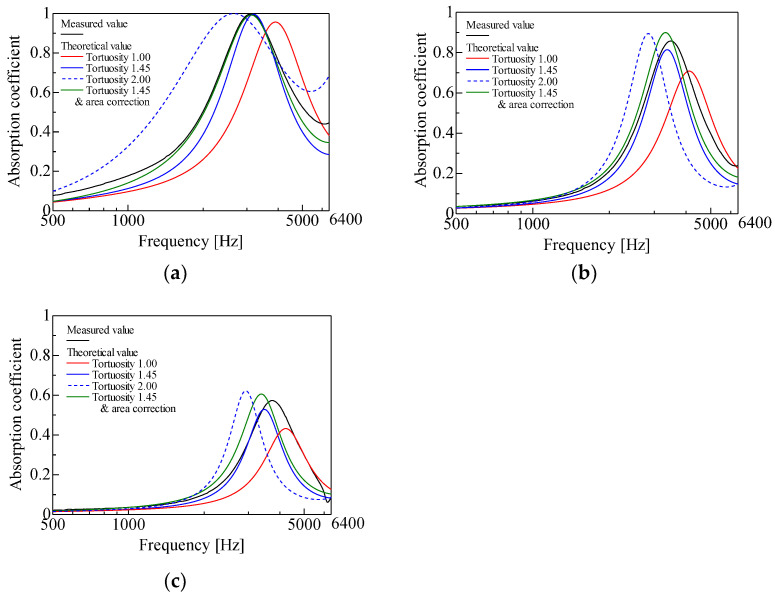
The experiment in comparison with calculation: (**a**) *d* = 1 mm; (**b**) *d* = 2 mm; (**c**) *d* = 4 mm.

The measured and theoretical values of the normal incident sound absorption coefficient were compared for each sample with a varying particle diameter in a random packing structure. First, the results were demonstrated, as shown in Figure 10a–c. Figure 10a–c show comparisons for the particle diameters *d* = 1 mm, *d* = 2 mm, and *d* = 4 mm, respectively.

First, a comparison was performed between the measured values in each graph and the theoretical values without considering the tortuosity. In all of the cases shown in Figure 10a–c, the theoretical values without considering the tortuosity appeared at a frequency higher than the peak frequency of the measured values. In all cases, the theoretical values without considering the tortuosity had lower peak sound absorption values than the measured values. The differences between the theoretical and measured values could be attributed to the fact that tortuosity was not considered.

Attention was then drawn to the theoretical values considering the tortuosity. Considering the tortuosity, the peak frequency moved lower in all cases, as shown in Figure 10a–c, and the peak sound absorption value increased in all cases.

As a result, the difference between the theoretical and measured values decreased when the tortuosity was considered. A tortuosity greater than unity means that the path of the sound waves propagating in the sample is longer, which has the same effect as an increase in the sample thickness. Thus, it can be considered that the peak frequency, considering the theoretical value, moved to the lower frequency side. For the same reasons mentioned above, the peak sound absorption values in the theoretical values could be considered to have increased due to the tortuosity consideration. The theoretical values for a tortuosity of 2.00 were also demonstrated for reference.

Theoretical values were considered with the surface area correction described in Section 4.2 after considering tortuosity. For the particle diameters *d* = 1 mm and *d* = 2 mm shown in Figure 10a,b, the theoretical and measured values for both the peak frequency and peak sound absorption agreed well when the tortuosity and surface area corrections were considered. The reason for this result was that the surface area correction reduced the void thickness *b*_n_ between the two planes in Equation (2), resulting in a larger proportion of the boundary layer in the voids and a larger calculated attenuation of the sound waves by viscosity. However, for the grain size *d* = 4 mm, as shown in Figure 10c, the peak frequency of the theoretical value was even lower than the measured value when considering the tortuosity and surface area corrections, and the reasons for this result are discussed below.

There are three possible reasons for the different degrees of agreement between the measured and theoretical values depending on the particle diameter. As a note, it is generally accepted that for equal packing structures, the paths of sound waves are analogous irrespective of the particle diameter; therefore, the same tortuosity can be applied. Thus, tortuosity is not considered the cause of the abovementioned results.

The first possible reason is that the method used to estimate the attenuation of sound waves in clearance [14] is more inaccurate for larger gaps (grain size) [16].

The second possible reason is the difference between the CT scan image and the sample used to measure the sound absorption coefficient. In the CT scan image, only a part of the particle was captured at the edge of the image, as shown in Figure 5a. In the experiment, the particles were spherical even at the edge, and there was a wall on the inner circumference of the sample tube. Thus, the difference between the CT scan image of the particles at the edge and the sample used for the measurement of the sound absorption coefficient could be a reason for the difference between the theoretical and measured values.

The third possible reason is an error in the layer thickness when the granular material was filled. To perform the experiment with a layer thickness of 20 mm, the granular material was added to the measuring tube and then slid off at a position of 20 mm from the rigid wall at the bottom of the measuring tube. Therefore, there were actually scattered cavities with a layer thickness of less than 20 mm. The effect of this phenomenon is more pronounced for larger particle diameters. In general, the smaller the sample thickness, the higher the peak frequency moves and the lower the peak sound absorption value. Therefore, in the case of a particle diameter of *d* = 4 mm in Figure 10c, it can be considered that the peak frequency of the measured value moved to a higher frequency due to levelling.

In summary, the obtained difference between the experimental and theoretical values (red line) was because the tortuosity and actual surface area were not considered. In other words, the obtained theoretical value based on both the tortuosity and actual surface area agreed well with the experimental value. The theoretical value that considered the tortuosity and surface area correction was less sharp (i.e., slightly higher than the experimental value at the base of the sound absorption curve). This result suggests that the attenuation of sound waves was slightly overestimated when considering the two factors. However, the policy of considering the tortuosity and surface area correction is suggested to be correct.

## 6. Conclusions

A theoretical calculation of the sound absorption coefficient at a normal incidence for the random packing of granular materials was performed using tomography images taken by CT scan. The measured tortuosity was considered in the derivation of the propagation constant and characteristic impedance. In the theoretical analysis, a surface area correction was performed to make the cross-sectional images closer to reality. Theoretical values with and without considering tortuosity, theoretical values with considering both tortuosity and surface area correction, and experimental values were compared, and the following results were obtained.

1. It is difficult to construct a mathematical model for random packing, as it has no structure periodicity. Therefore, the sound absorption coefficient was estimated using a theoretical analysis based on cross-sectional CT scan images.
2. For the theoretical values considering the tortuosity, the peak sound absorption values were higher, and the peak frequency moved to lower frequencies compared with the case without considering the tortuosity. As a result, in all cases, the theoretical values were closer to the measured values. Therefore, the measured tortuosity values are reasonable.
3. Regarding the theoretical values, when both the surface area and tortuosity were considered, the peak sound absorption frequency moved to a lower frequency compared with the theoretical value when only considering tortuosity, and was in general agreement with the measured values for the particle diameters of *d* = 1 mm and *d* = 2 mm. Therefore, the method of estimating the vertical incident sound absorption coefficient using computed tomographic images is useful. Moreover, this model can be applied even if the material changes, provided the granular material can be considered rigid. Additionally, the model can be applied without problems for general granular densities and grain sizes in the order of mm.

## Figures and Tables

**Table 1:** Result of the tortuosity measurement.

Frequency [kHz]	Inverse of the Square Root of Frequency $\left[1/\sqrt{Hz}\right]$	Distance between Sensors [mm]	Transmission Time [ms]	Tortuosity
32.7	0.00559	395	1.229	2.09
58	0.004152	395	1.199	1.53
110	0.003015	345	1.044	2.13
150	0.002582	204	0.626	2.00
200	0.002236	204	0.618	1.67
300	0.001826	204	0.615	1.33
∞	0	-	-	1.45
